# The Involvement of Academic and Emotional Support for Sustainable Use of MOOCs

**DOI:** 10.3390/bs14060461

**Published:** 2024-05-30

**Authors:** Zhanni Luo, Huazhen Li

**Affiliations:** School of Foreign Languages and Literatures, Chongqing Normal University, Chongqing 401331, China; 2021210506033@stu.cqnu.edu.cn

**Keywords:** MOOCs, engagement, dropout rate, academic support, emotional support, platform reputation, technology acceptance, EFL learning

## Abstract

MOOCs, the Massive Open Online Courses, are online educational courses that offer open access to a large number of participants globally. However, online engagement during MOOC learning remains a problem, as reflected in relatively high dropout rates. This paper involves academic and emotional support, aiming to explore whether they contribute to users’ sustainable use of the MOOC platform. A total of 410 college students learning English as a foreign language (EFL) and with MOOC learning experience participated in this study. Employing the structural equation modeling (SEM) techniques, we examined the relationships among five factors in the EFL MOOC learning context: academic support (AS), emotional support (ES), perceived usefulness (PU), perceived ease of use (PEoU), and platform reputation (PR). The results indicate that academic support influences learners’ perceptions of the usefulness and ease of use of the MOOC platform, as well as enhancing learners’ feelings of being emotionally supported. Simultaneously, platform reputation plays a crucial role in influencing learners’ perceptions of MOOC platforms. However, results suggest that emotional support does not have a statistically significant impact on the perceived usefulness and perceived ease of use of the platform in EFL MOOC learning contexts.

## 1. Introduction

### 1.1. MOOCs and MOOC Platforms: Definition, Importance, and Crisis Regarding Sustainable Development

Distance education, characterized by its ubiquity, enables learners to access high-quality educational resources without constraints [1]. Accordingly, over the past two decades, distance education has experienced rapid development. The global COVID-19 pandemic prompted most countries to implement measures facilitating home-based learning, further expediting the evolution of distance education [1]. Among the various forms of distance learning, the Massive Open Online Courses (MOOCs) is a well-recognized one.

MOOCs, the Massive Open Online Courses, are internet-based courses designed for a large number of participants [2]. MOOC platforms are online learning environments that offer massive open online courses, such as Coursera, edX, and Udacity. These platforms provide a wide range of courses from various disciplines, often in partnership with universities and colleges, enabling accessible education for anyone with an internet connection. MOOC platforms also incorporate multimedia elements like video lectures, quizzes, and forums for an engaging learning experience. Originating in response to the demand for accessible education, MOOCs have gained prominence for breaking geographical barriers and allowing learners diverse access to quality content globally [3]. The global reach of MOOCs has transformed education, inspiring innovative and inclusive knowledge-sharing approaches among educators and learners.

### 1.2. The Involvement of Academic and Emotional Support: Gaps, Significance, and Research Focuses

However, a critical failure of MOOCs has been that participants’ engagement in MOOCs is highly irregular and self-directed, leading to a consistently high dropout rate [4,5,6,7,8,9,10,11]. The dropout rate is highly correlated with unsatisfactory academic performance, which continues to impact the MOOC platform’s reputation [12]. For learners, unsatisfactory engagement affects their sustained use of the MOOC platform. For the MOOC platform, the failure to retain learners also brings negative influences on its sustainable development. Therefore, MOOC learning should consider factors beyond knowledge delivery in conventional MOOC courses.

Researchers have discussed factors affecting learners’ intention to accept MOOC platforms, such as task-technology fit, social recognition, relative advantages, and individual openness [13,14,15]. However, few of them paid attention to the academic and emotional support provided by MOOC platforms. Wong [16] suggests that when designing or evaluating an educational technology, one should not only consider its attributes as a technology but also take into account two factors: its usefulness in improving learning outcomes and its ability to enhance the learning experience during the learning process. In the context of MOOC-based EFL learning, the corresponding two factors can be academic support and emotional support. Therefore, this paper posits that these two factors (academic support and emotional support) can provide learners with more assistance and feedback, helping MOOC learning to compensate for its deficiencies in purely focusing on knowledge delivery.

Therefore, we aim to involve academic and emotional support for sustainable EFL MOOC learning. Considering that technology acceptance is a prerequisite for the sustainable use of MOOC platforms, we also involved elements related to technology acceptance, including perceived usefulness (PU), perceived ease of use (PEoU), platform reputation (PR), and behavioral intention (BI). However, in our pilot study, the survey items measuring behavioral intention did not pass the reliability and validity test, so the corresponding items were deleted. Therefore, in the end, we verified the relationship between five factors. Eventually, we sought to answer four research questions (RQs) below:

RQ 1: What is the impact of academic support (AS) on learners’ perceived usefulness (PU) and perceived ease of use (PEoU) toward MOOC platforms?

RQ 2: What is the impact of emotional support (ES) on learners’ perceived usefulness (PU) and perceived ease of use (PEoU) toward MOOC platforms?

RQ 3: Is there a correlation between academic support (AS) and emotional support (ES) in MOOC learning?

RQ 4: Does the platform reputation (PR) of MOOCs positively influence academic support (AS) and emotional support (ES)?

## 2. Literature and Research Hypotheses

### 2.1. Transitions: From xMOOCs, cMOOCs, to Wrapped MOOCs

Scholars argue that one of the reasons for the high dropout rate in MOOCs is a lack of interaction, a key factor influencing learning engagement [3,17,18]. Consequently, the concept of MOOCs has been further classified into xMOOCs and cMOOCs [19].

Extended MOOCs, commonly known as xMOOCs, typically embrace a more traditional approach to online education [14]. They emphasize structured content delivery using methods like video lectures, self-assessment quizzes, and other carefully curated learning resources [20].

In contrast, cMOOCs focus on collaborative interactions among participants rather than a centralized teaching approach. cMOOCs encourage learners to build and share knowledge through online networks, fostering a more decentralized and participatory learning experience [21,22]. The shift from xMOOCs to cMOOCs signifies educators’ awareness that the mere provision of instructional videos is insufficient and that education requires more learner-centric interactions.

In the cMOOCs model, the challenge of insufficient engagement persists. In response, a more collaborative and interactive approach has emerged: the integration of MOOCs with a traditional face-to-face course, commonly known as wrapped MOOCs [23]. In the wrapped-MOOC model, online content and resources from MOOCs are seamlessly blended with in-person instruction [24]. This approach allows students to both access worldwide instructional videos and participate in teacher-student and student-student interactions [25]. As emphasized by Jaffer, Govender and Brown [26], the most valuable aspect of wrapped MOOCs is the face-to-face interactions.

From the above, it can be seen that interactions are important factors influencing the sustained use of MOOCs, and they are also something that MOOC platforms are actively working to strengthen [10]. However, it is worth noting that what exactly is provided by these interactions has not been clearly defined.

### 2.2. MOOCs and Technology Acceptance

MOOCs, with their distinct advantages, have experienced swift growth in the education field. Measuring technology acceptance is essential for predicting the success of a new technology, as the potential of even the most cutting-edge and feature-rich technology may remain untapped if it does not achieve broad acceptance among users [16,27,28]. Moreover, considering MOOCs’ challenges in maintaining learner retention, the intention of users to accept these platforms is especially critical.

A wide array of theoretical frameworks exists to unravel the complexities of technology acceptance. These include well-known models like the Technology Acceptance Model (TAM), which underscores factors like perceived ease of use and perceived usefulness [29]. The Unified Theory of Acceptance and Use of Technology (UTAUT and UTAUT2) delves into aspects such as performance expectancy, effort expectancy, social influence, and facilitating conditions, offering a comprehensive understanding of user engagement with technology [30,31]. Additionally, the Innovation Diffusion Theory by Rogers [32] provides a thorough exploration of the mechanisms through which new technologies and ideas permeate cultures, examining the dynamics of adoption over time and across different social groups. Each of these models contributes significantly to our understanding of the complex interplay of factors that drive users towards or away from adopting new technologies, highlighting the multifaceted nature of technological acceptance and integration.

Among the numerous studies exploring the relationship among contributing factors to technology acceptance, some have reported unexpected findings. For example, Wu and Chen [14] attempted to integrate the TAM model with the Task Technology Fit (TTF) model. They hypothesized that if MOOCs are perceived as easy to use or can bring about social influence, learners would have a better attitude toward MOOCs. However, the results showed that these two hypotheses were not supported. Al-Rahmi, Yahaya, Alamri, Alyoussef, Al-Rahmi and Kamin [13] integrated the TAM model with the Innovation Diffusion Theory (IDT). They hypothesized that if MOOCs were perceived as highly compatible, they would be perceived as useful, and if MOOCs had trialability, they would be perceived as easier to use. These two hypotheses were also not supported, neither.

The unexpected findings and the unsupported hypotheses indicate a need for further research in the field of technology acceptance, especially concerning the integration of different theoretical models and their applicability in varied contexts like MOOCs.

### 2.3. Perceived Usefulness, Perceived Ease of Use, and Technology Acceptance Intention

In the field of technology acceptance, a well-established model is the TAM model by Davis, Bagozzi and Warshaw [29]. The TAM model indicates that the main factors influencing an individual’s attitude and intention to accept a technology are perceived usefulness and perceived ease of use.

Perceived usefulness is defined as the degree to which an individual believes that using a particular system would enhance his or her job performance [29]. According to previous studies, perceived usefulness has a positive effect on technology acceptance intention [33,34,35,36].

Perceived ease of use is defined as the degree to which an individual believes that using a particular system would be free of physical and mental effort [29], which is similar to effort expectancy in the unified theory of acceptance and use of technology (UTAUT) framework [31]. Results from the extant literature indicate that perceived ease of use was a statistically significant predictor of intention to use new technologies, such as the Internet and educational games [35].

There is a correlation between PEoU and PU because Davis, Bagozzi and Warshaw [29] indicates that PEoU should be a contributing element to PU. In other words, individuals are inclined to believe that a technology is useful if they perceive it as easy to use. Therefore, we established the first hypothesis (H1):

**H1.**  *The perceived ease of use (PEoU) of MOOCs positively influences the perceived usefulness (PU) of MOOCs.*

### 2.4. Academic Support in MOOC Learning

Academic support, also named instructional support, refers to “instructional guidance to learning, which involves answering students’ questions, correcting their misunderstandings, providing clear instruction, relevant resources, and constructive feedback on their assignments and performance” [37]. Academics are aware of the importance of academic support in online education and advocate to ensure its provision [37]. In the xMOOCs model, sufficient academic support can hardly be guaranteed.

Firstly, to maintain user attention, instructional videos on MOOC platforms are often kept relatively short, typically ranging from 5 to 17 min [38]. This feature, accordingly, limits the academic depth of MOOC learning resources.

Secondly, on MOOC platforms, teachers and students do not physically appear simultaneously, making real-time interaction impossible [39]. Although some MOOC platforms may include interactive channels such as discussion forums and scheduled Q&A sessions, the opportunities for teachers to provide feedback are limited [3]. Previous studies have also indicated that certain posts in MOOC discussion forums require an urgent response, but instructors find it challenging to attend to such situations [9]. Therefore, some researchers have even attempted to establish help-seeking mechanisms in MOOCs [10]. These efforts demonstrate that academic support on MOOC platforms is not always sufficient, particularly concerning learning Q&A and learning feedback.

Moreover, due to technological and financial constraints, MOOC platforms are not able to provide personalized learning materials to learners. Most MOOC platforms offer one-size-fits-all content to all learners, making it difficult to provide sufficient support for everyone [2,40]. Nowadays, researchers are attempting to address the lack of personalized learning by incorporating artificial intelligence (AI) and chatbots into MOOC platforms [41,42,43,44]. These efforts further demonstrate that simply presenting short videos on MOOC platforms is insufficient.

As the content on MOOC platforms may lack depth and flexibility, the academic support provided by teachers and peers becomes crucial. Students require more detailed explanations from teachers to help them better grasp knowledge [45,46], as well as feedback from both teachers and peers tailored to their learning [47]. We can hypothesize that if the design of a MOOC platform allows teachers and peers to offer enhanced academic support to students, the MOOC platform is more likely to be accepted by students. This hypothesis is also applicable to learning English as a foreign language on MOOC platforms. Therefore, we hypothesized that academic support positively influences EFL learners’ behavioral intention towards MOOC platforms.

As stated in the Section 1, in our pilot study, the survey items intended to measure behavioral intention did not pass reliability and validity tests, leading to their removal. Consequently, we did not formulate a hypothesis regarding the impact of academic support on the behavioral intention of MOOC platforms. Instead, we focused on its influence on perceived usefulness and perceived ease of use of MOOC platforms:

**H2-a.**  *Academic support (AS) positively influences the perceived usefulness (PU) of MOOCs.*

**H2-b.**  *Academic support (AS) positively influences the perceived ease of use (PEoU) of MOOCs.*

### 2.5. Emotional Support in MOOC Learning

Emotions play a crucial role in students’ learning, as they can either hinder or inspire the learning process [46,48]. Emotional support, in general, involves providing empathy, concern, affection, love, trust, acceptance, intimacy, encouragement, or caring [49]. In the context of using MOOCs for learning, emotional support primarily comprises encouragement that convinces students of the usefulness of MOOCs and their capability to master the relevant courses, ultimately leading to a stronger sense of self-efficacy. When students feel isolated in the online environment, the providence of emotional support can also alleviate the sense of loneliness.

In research related to MOOCs, emotional isolation is a frequently mentioned term. Vilkova and Shcheglova [10] state that MOOC platforms provide a self-paced, open-ended, and non-linear learning environment, which can help cultivate students’ learning autonomy. However, this also requires students to take responsibility for their own learning process. In this process, students are prone to developing feelings of emotional isolation, making it easier for them to disengage from the course [10,18,50].

Although many studies suggest that learners can reduce emotional isolation through the discussion forum [47], relying solely on discussion forums may not be sufficient. Vilkova and Shcheglova [10] emphasized that despite the widely accepted importance of interactions and social engagement, “a MOOC environment does not allow participants to interact easily with other learners and instructors”. This significantly restricts students’ ability to receive emotional support. Additionally, some MOOC platforms send emails to students when there is new information in the discussion forum, but certain students may post meaningless questions. This can lead to students receiving a large number of spamming emails, further diminishing their trust in the MOOC platform and making them hesitant to participate in the discussion forum.

Discussion forums may also fail to provide emotional support for certain types of learners. Researchers noted that low-achieving students tend not to engage in peer interactions or discussions but prefer learning from their peers’ discussions [18]. Based on the Felder–Silverman Learning Style Model (FSLSM), researchers highlight that learners have different types, with some prefer learning through participation in discussion forums (active learners), while some prefer processing information internally, indicating a preference for observing information generated by others (reflective learners) [40,51]. If these students need interaction with teachers and peers on discussion forums for emotional support, not getting enough of it could lead to disengagement or even dropout of MOOC courses.

Offering emotional support through alternative channels, specifically excluding discussion forums, is possible. In instructional videos, teachers can consciously incorporate eye contact with potential learners, increase the use of praise in classroom language, and use encouraging tones [27]. The MOOC platforms can incorporate game elements such as points, badges, leaderboards, sound effects, etc., to make students feel emotionally cared for [36,52]. The MOOC platform can also generate learners’ progress reports, enabling learners to share them on their social networking sites (e.g., Twitter, Facebook, WeChat) and receive emotional support through peers’ comments and likes [53].

Researchers argue that typical xMOOC models primarily focus on didactic education, emphasizing information delivery [22]. When students perceive less emotional support, negative emotions may arise, such as frustration, confusion, boredom, and emotional isolation [54]. These emotions experienced by online learners may hinder the learning process and fail to provide sufficient intention or motivation for students to persist [55]. Building on this literature, we propose the following two hypotheses:

**H3-a.**  *Emotional support (ES) positively influences the perceived usefulness (PU) of MOOCs.*

**H3-b.**  *Emotional support (ES) positively influences the perceived ease of use (PEoU) of MOOCs.*

Some research has implied that high perceptions of academic quality positively influence the PU of online education. To be specific, Alraimi, Zo and Ciganek [56] stated that academic support influences the online learning outcomes of students. Accordingly, we establish the fourth hypothesis (H4):

**H4.**  *Academic support (AS) positively influences emotional support (ES) provided by MOOCs.*

### 2.6. Platform Reputation and Technology Acceptance

The role of reputation has been highlighted in different fields [56]. Notable platforms like Coursera and edX offer high-quality courses by partnering with highly reputable institutions and universities from different countries [56]. The reputation of an institution of higher education is a subjective reflection of the institution’s quality, influence, and trustworthiness [57]. Reputation is a valuable and intangible asset (Tella et al., 2021), which has a significant influence on an individual’s decision-making process when selecting institutions or MOOC platforms [57,58]. Researchers have found a significant and positive impact of the perceived reputation of MOOCs on users’ acceptance intention [14,56].

Researchers also indicate that perceived usefulness and perceived ease of use are significantly and positively influenced by platform reputation [14,59]. MOOC platforms with a high reputation usually have more abundant funds and development teams, enabling their MOOC platforms to achieve better development in educational attributes and technological operational convenience. We can also hypothesize that if a MOOC platform can provide better academic and emotional support, its platform reputation will also be enhanced. Therefore, we propose the following hypotheses:

**H5-a.**  *In MOOC learning, platform reputation (PR) positively influences academic support (AS).*

**H5-b.**  *In MOOC learning, platform reputation (PR) positively influences emotional support (ES).*

**H5-c.**  *In MOOC learning, platform reputation (PR) positively influences perceived usefulness (PU).*

**H5-d.**  *In MOOC learning, platform reputation (PR) positively influences perceived ease of use (PEoU).*

**Figure 1:**
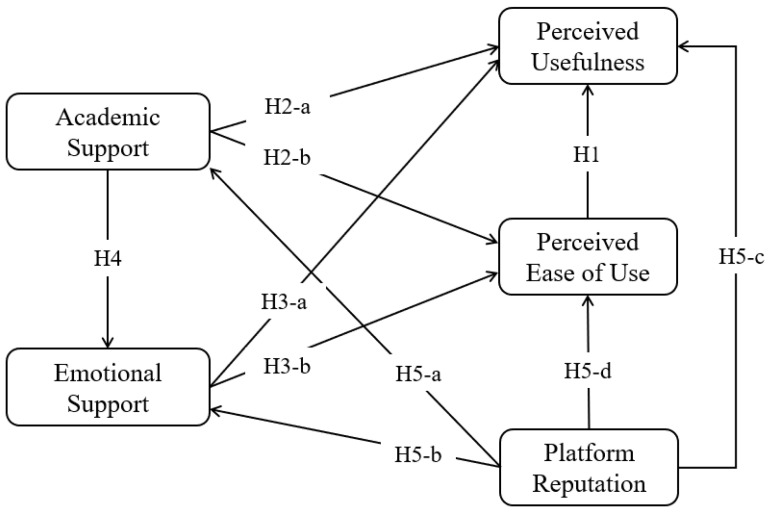
The proposed framework.

The theoretical framework and hypotheses are depicted in Figure 1.

## 3. Methodology

### 3.1. Research Instrument

The instrument utilized in this study comprised two parts. The part, named “demographic information”, focused on gathering fundamental details about the participants, encompassing gender and grade. The second part, named the “online MOOC learning scale”, consisted of five subscales measuring academic support, emotional support, platform reputation, perceived usefulness, and perceived ease of use.

The subscales measuring perceived usefulness and perceived ease of use were adapted from Davis, Bagozzi and Warshaw [29]. The former subscale measures how much a person believes that using a system will improve their job performance, while the latter subscale measures how easy a person thinks the technology is to use. Each subscale includes three items.

The emotional support subscale was adapted from Jiang and Koo [60], which includes three items. This subscale measures the degree of emotional support that students perceive from their teachers. For example, one item states: “Teachers will encourage me to express my views in my program of study”. This item reflects the supportive environment that teachers create, allowing students to feel comfortable sharing their opinions and participating actively in their education [60].

The academic support subscale was adapted from the Students’ Perception of Support and Course Satisfaction Scale, which helps to understand the help and guidance students receive from the teacher while taking the course in MOOCs [61]. For example, one item states: “The instructor gives timely feedback on the assignments I submit to the MOOC platform”.

The platform reputation was designed based on Kim, Ferrin and Rao [62], which includes four items. This subscale evaluates the perceived reputation of the MOOC platform. For instance, one item states: “Prestigious universities are involved in MOOCs”. This item reflects the platform’s credibility and the high regard in which it is held, which can significantly influence students’ trust and their decision to engage with the courses offered.

We attempted to use the survey items as they were, only changing the subject, such as replacing “the technology” with “MOOCs”. This approach aims to maintain the reliability and validity of the surveys as much as possible, as well as readability.

We have also incorporated two filter items to ensure response accuracy. The first item asks, “Have you ever used MOOCs to learn English?” If the answer is no, the questionnaire is deemed invalid. The second one instructs participants to select “Disagree” for the item. In cases where participants predominantly choose “Agree” or “Strongly Agree” for other questions, selecting “Disagree” indicates a deliberate response, rather than a habitual or perfunctory selection.

All items, except for the two demographic questions, were measured using a 5-point Likert scale, ranging from 1 (strongly disagree) to 5 (strongly agree).

### 3.2. Data Collection and Participants

After determining the questionnaire content, we employed a professional translator to translate it into Chinese. Then, we employed the back-translation technique to ensure translation accuracy. Once the questionnaire was finalized, we transformed it into a digital format and distributed it to college students using the convenience sampling method. The duration for the data collection was eight months.

In the end, we collected a total of 595 surveys. Among them, 85 surveys indicated that the respondents had no experience using MOOCs to learn English, and 100 surveys were invalid because the participants chose other options when they were required to select “Disagree”. Consequently, we obtained 410 valid survey responses.

Among these 410 participants, all were above the age of 18, with a significant majority being female (n = 254, 62%). There were 351 undergraduate students (85.6%) and 59 master’s students (14.4%) (see Table 1). All participants were aware of the research intentions and signed the consent form. Participation was completely anonymous and voluntary.

### 3.3. Data Analysis

The data analysis utilized the software SPSS 26 and AMOS 26. Initial steps involved Confirmatory Factor Analysis (CFA), assessing reliability, and establishing validity to evaluate the subscales’ fit, stability, and effectiveness. Subsequently, descriptive analysis and correlation analysis were performed to explore the foundational characteristics of the subjects. Finally, Structural Equation Modeling (SEM) was employed to examine the interrelationships among the six constructs.

## 4. Findings

### 4.1. Descriptive Statistics

Table 2 reports the mean values, standard deviation values, skewness, and kurtosis of the selected five variables. The data reveals that the mean scores for all variables are relatively high, ranging from 3.75 to 4.01 on a scale, indicating overall positive perceptions among the respondents. The data distribution is consistently left-skewed, with some values showing moderate skewness and others indicating a higher degree of skewness. This implies that the majority of respondents chose higher values, with fewer lower values stretching out towards the left.

### 4.2. Reliability and Validity Analysis

The data analysis demonstrates the suitability of this dataset for factor analysis. This is evidenced by the Kaiser–Meyer–Olkin (KMO) measure, which stands at 0.907, significantly surpassing the generally accepted minimum of 0.6. This high KMO value indicates that the dataset has adequate sampling adequacy. Moreover, Bartlett’s Test of Sphericity shows a chi-square value of 4286.699 with a significance level less than 0.001, strongly indicating that the variables are interrelated, thereby validating the applicability of factor analysis for this dataset.

The factorial analysis conducted using Principal Component Analysis with Promax rotation provides a comprehensive assessment of the constructs in the study. The constructs exhibit high factor loadings for each survey item, indicating a strong alignment of the items with their respective constructs. The factor loadings range from 0.830 to 0.924, demonstrating a robust correlation between the items and their factors (see Table 3).

The total variance explained by these factors is an impressive 79.297%, suggesting a strong model fit and the effectiveness of the constructs in explaining the variability in the data. The breakdown of this variance across the different factors, with Platform Reputation accounting for the highest variance, highlights the differential impact of each construct on the overall model.

The reliability and validity of the constructs are further evidenced by the high Cronbach’s Alpha values (ranging from 0.850 to 0.940), indicating excellent internal consistency. Additionally, the Average Variance Extracted (AVE) for each factor surpasses the 0.5 threshold, and the Composite Reliability (CR) scores exceed 0.9, which collectively affirm the reliability and construct validity of the measurement model.

The strong positive correlations between AS and ES (0.561 **), as well as between PR and PU (0.599 **), imply that these factors are interdependent. This suggests that when students perceive high academic and emotional support, they also tend to view the platform as more reputable and useful. Similarly, the positive correlation between PR and PEoU (0.528 **) indicates that a platform’s reputation may play a crucial role in how easily users feel they can utilize it.

The square roots of AVE (ranging from 0.868 to 0.904) exceed the inter-construct correlations, indicating discriminant validity. Furthermore, the high correlations (indicated by) between the constructs, such as between AS and ES (0.561), and between PR and PU (0.599 **), suggest a strong interrelationship, reinforcing the relevance and interconnectedness of these variables in the context of the study (see Table 4).

### 4.3. Model Fit

We utilized several model fit indices, including CMIN/df (chi-square to degrees of freedom ratio), CFI, GFI, AGFI, NFI, TLI, and RMSEA, to assess the adequacy of the model. The full names of each index are detailed in Table 5. The chi-square to degrees of freedom ratio for the model in this study is 1.851, falling within the range of greater than or equal to 1 but less than 3, thus meeting the criteria for “excellent”. The performance of this model in other model fit indices also aligns with the “excellent” category, as shown in Table 5.

These values collectively indicate a strong fit of the collected data with the measurement model, aligning with the principles established by Bentler and Bonett [63], Hu and Bentler [64], and MacCallum, Browne and Sugawara [65].

### 4.4. Hypothesis Testing

The results indicate that eight out of ten hypotheses have been confirmed. Consistent with the assumptions proposed by Davis, Bagozzi and Warshaw [29] when introducing the Technology Acceptance Model, in EFL MOOC learning contexts, the PEoU of MOOCs promotes the PU of MOOCs (*β* = 0.209, *p* < 0.001). In other words, if learners find the MOOC platform easy to use, they are likely to perceive it as useful (H1). Academic support, recognized as a critical necessity for learners, significantly influences their perceptions of both PU (*β* = 0.127, *p* < 0.05) and PEoU (*β* = 0.189, *p* < 0.01) of the MOOC platform, thereby supporting H2-a and H2-b. This indicates that learners who receive adequate academic support are more likely to find the MOOC platform useful and user-friendly. Moreover, academic support contributes to learners experiencing a greater sense of emotional support (*β* = 0.080, *p* < 0.001), as confirmed in H4.

Meanwhile, in the context of EFL MOOC learning, platform reputation plays a pivotal role in influencing learners’ perceptions. As demonstrated in Table 6, when learners have a positive perception of the MOOC platform’s reputation, they are more likely to believe that they will receive substantial academic support (H5-a) (*β* = 0.672, *p* < 0.001) and emotional support (H5-b) (*β* = 0.283, *p* < 0.001). Additionally, a positive platform reputation enhances learners’ perceptions of the MOOC platform’s usefulness (H5-c) (*β* = 0.427, *p* < 0.001) and technical ease of use (H5-d) (*β* = 0.469, *p* < 0.001).

However, H3-a and H3-b were not validated. The results indicate that emotional support does not have a significant impact on the perceived usefulness (*p* = 0.160) or the perceived ease of use (*p* = 0.136) of the MOOC platform. This means that providing emotional support does not directly affect how useful or easy to use learners find the MOOC platform. The reasons for these unexpected results are unclear and need further discussion and validation (see Table 6). Future research should explore why emotional support does not influence these perceptions to better understand this relationship.

## 5. Discussion

In this study, when investigating the factors influencing learners’ acceptance intention to use MOOCs for EFL learning, we integrated academic support, emotional support, and platform reputation into the TAM model. Three main factors in the TAM model were used, including PU, PEoU, and behavioral intention. However, the items measuring behavioral intention failed to pass the scale validation in the pilot study, resulting in the removal of the “behavioral intention” construct from the model. This finding is inconsistent with the theory [35,66].

One possible reason is that the other constructs involved in this study are more closely related to the MOOC platform itself, such as the academic support provided by the MOOC platform and the perceived usefulness of the MOOC platform, while behavioral intention focuses on users (rather than the MOOC platform). Another explanation is that the perceived benefits of a technology do not necessarily align with users’ willingness to use it [27]. There are various factors influencing users’ intention to use technology, such as a lack of facilitating conditions [31], a lack of necessary knowledge [16,27,67], a lack of relatedness [35,52], negative attitudes towards the technology within their social circles [30], concerns about privacy and cybersecurity issues [68], and users not being entirely voluntary in their use of the technology [30].

Among the two unconfirmed hypotheses, one is H3-a: emotional support (ES) positively influences the perceived usefulness (PU) of MOOCs. Luo, Brown and O’Steen [27] suggest that to evaluate the usefulness of an educational tool, one should not only consider its usefulness in terms of academic improvement but also its usefulness in enhancing learners’ learning experience. Therefore, if learners can receive sufficient emotional support on the MOOC platform, their learning experience will be better, implying a better perceived usefulness of the educational tool. The data indicates that ES and PU do not have a statistically significant correlation, thus not supporting the above hypothesis. One possible explanation is that the emotional support provided via MOOC platforms is insufficient, leading to an inability to enhance the learning experience. Another possible hypothesis is that the items measuring PU are still too focused on learning outcomes rather than the learning process [35]. The underlying reasons still need further exploration.

Another unconfirmed hypothesis is H3-b: emotional support (ES) positively influences the perceived ease of use (PEoU) of MOOCs. Similar to the previous hypothesis, if the emotional support provided through MOOC platforms is inadequate, learners may not perceive it as a significant factor in making the MOOC learning process perceived easier. The PEoU of the MOOC platform is also influenced by contextual factors such as the nature of the course content, the design of the MOOC platform, or the learners’ prior familiarity with online learning environments [69]. At the same time, individual differences can also influence the results because different learners may have diverse needs for emotional support, and they possess varying digital competencies, leading to differences in the perceived ease of use of the MOOC platform [70]. Additionally, as in other studies exploring the relationships between factors, there may be other unexplored factors influencing the perceived ease of use of the MOOC platform within the context of EFL MOOC learning. Therefore, the relationship between emotional support and perceived ease of use can be complex [52]. While the mentioned reasons can partially explain this unconfirmed hypothesis, specific details require further rigorous validation.

Overall, this study emphasizes the importance of providing emotional support in online learning environments. Learner emotions have been largely neglected for a long time, yet research has found that emotions can indeed predict academic performance [71]. This growing emphasis on the role of emotions in learning has led scholars to explore different dimensions of emotional support and engagement in online learning. Specifically, scholars have attempted to categorize the perceived usefulness of technology into two main concepts: the perceived usefulness of improving outcomes and the perceived usefulness of improving the learning experience [16,72]. This division highlights the importance of focusing on the emotional well-being and satisfaction of learners during the learning process. Furthermore, scholars have differentiated engagement into behavioral engagement, emotional engagement, and cognitive engagement [73,74]. Emotional engagement, also named affective engagement, captures learners’ emotional responses, attitudes, and feelings toward the learning process [8,27]. This distinction recognizes that learners’ emotions play a crucial role in shaping their engagement and motivation, ultimately affecting their learning outcomes.

Although this study indicates that emotional support provided by MOOC platforms has not positively influenced the MOOC platform’s perceived usefulness and perceived ease of use, it suggests that other factors may enhance learners’ emotional well-being on MOOC platforms. Specifically, scholars are suggested to consider the determinants of emotional well-being, rather than focusing solely on the impact of emotional support as a factor.

## 6. Conclusions

This study explores the role of academic and emotional support in sustaining users’ engagement with MOOCs (Massive Open Online Courses). The study involves 410 college students learning English as a foreign language (EFL) through MOOCs, examining the relationships among factors such as academic support, emotional support, perceived usefulness, perceived ease of use, and platform reputation. Two out of ten hypotheses were not confirmed, suggesting that emotional support does not have a statistically significant impact on the perceived usefulness and perceived ease of use of the platform in EFL MOOC learning.

One limitation is that, despite using a validated survey with no technical issues, the content of the survey was somewhat simplistic. Wong [16] suggests that perceived usefulness in educational contexts can be divided into perceived usefulness regarding learning outcomes and perceived usefulness regarding the learning process. Luo [35] further expands perceived usefulness to include curriculum relatedness and social influence. That is, when accepting educational technology, various factors influence learners’ willingness, including the perceived usefulness in improving learning outcomes (PU-outcome), the perceived usefulness in enhancing the learning experience (PU-process), curriculum relatedness, and the societal impact of using the technology (e.g., feeling respected). Luo, Tan, He and Wu [72] used grounded theory to expand the determinants of perceived usefulness in the context of using interactive whiteboards for EFL learning. The results of Luo et al. (2023) indicate that the concept of perceived usefulness can indeed be enriched. Future research could build on this study by using PU scales that involve more constructs [28].

Future research can further understand how learners’ motivations for studying English on MOOC platforms dynamically change, identify factors contributing to motivation changes, and explore ways to enhance students’ learning motivation [50]. Since discussion forums are important channels for learners to receive academic and emotional support from teachers and peers, future research could investigate how can we utilize discussion forums to facilitate peer interactions, affective engagement, cognitive engagement, and academic performance [3,20,47,52].

Given that MOOC platforms can capture extensive learner behavior data, future research can analyze this data for more accurate instructional support, or even personalized learning experiences [9,20]. Additionally, for better engagement maintenance, it could be meaningful to integrate gamification techniques in MOOC platforms [27,35,52]. Overall, the sustainable development of MOOC platforms requires more academic and emotional support. Future studies could explore how to provide support on MOOC platforms, what type of support is needed, and how effective the support provision is.

## Figures and Tables

**Table 1:** Demographic information of the participants (sample size = 410).

Demographic Variable		Number	Percentage
Gender	Male	156	38
	Female	254	62
Educational status	Undergraduate	351	85.6
	Postgraduates	59	14.4

**Table 2:** Descriptive statistics.

Construct	N	Mean	Std. Deviation	Skewness	Kurtosis
Academic support (AS)	410	3.75	0.997	−0.797	0.065
Emotional support (ES)	410	3.81	0.919	−0.826	0.558
Platform reputation (PR)	410	4.01	0.849	−1.035	1.208
Perceived usefulness (PU)	410	3.97	0.897	−1.085	1.15
Perceived ease of use (PEoU)	410	3.81	0.893	−0.69	0.27

**Table 3:** Survey items and the exploratory factor analysis.

Construct	Item Code	Survey Item	Factor/Factor Loading				
			1	2	3	4	5
Academic Support	AS1	The instructor or peers give timely feedback on the assignments I submit to the MOOC platform.		0.924			
	AS2	I could ask questions regarding the course materials on the MOOC platform.		0.906			
	AS3	My peers on the MOOC platform are willing to provide academic help.		0.880			
Emotional Support	ES1	The instructor encourages me to express my views in the coursework in MOOC study.			0.888		
	ES2	The instructor recognizes my completion of the MOOC courses.			0.869		
	ES3	The instructor gives me suggestions and advice that will help me build up confidence during my MOOC learning.			0.895		
Platform Reputation	PR1	The MOOC platform has a high reputation.	0.912				
	PR2	The universities that offer courses on the MOOC platform have high reputation.	0.830				
	PR3	The MOOC platform is widely recognized.	0.896				
	PR4	The instructors teaching on the MOOC platform are widely recognized.	0.832				
Perceived Usefulness	PU1	The MOOC platform provides me with more learning resources.					0.839
	PU2	The MOOC platform increases my learning efficiency.					0.904
	PU3	The MOOC platform makes my learning more convenient.					0.864
Perceived Ease of Use	PEoU1	It is easy to use MOOC platform for learning.				0.834	
	PEoU2	Overall, using the MOOC platform is simple.				0.914	
	PEoU3	I can quickly master the use of MOOC platform for learning.				0.853	
Total Variance Explained: 79.297%			47.37%	10.71%	7.91%	6.80%	6.51%
Cronbach’s Alpha (*α*): 0.940			0.899	0.897	0.871	0.850	0.866
AVE (Average Variance Extracted)			0.754	0.817	0.782	0.752	0.756
CR (Composite Reliability)			0.924	0.93	0.915	0.901	0.903

**Table 4:** Correlation and the square root of AVE.

Construct	AVE	Square Root of AVE	Correlation				
			AS	ES	PR	PU	PEoU
Academic support (AS)	0.817	0.904	1				
Emotional support (ES)	0.782	0.884	0.561 **	1			
Platform reputation (PR)	0.754	0.868	0.492 **	0.467 **	1		
Perceived usefulness (PU)	0.756	0.869	0.485 **	0.441 **	0.599 **	1	
Perceived ease of use (PEoU)	0.752	0.868	0.450 **	0.399 **	0.528 **	0.508 **	1

The table shows the square root of AVE on the diagonal and correlations between the latent constructs on the off-diagonal. ** *p* < 0.01.

**Table 5:** Model fit indices.

Model Fit Index		Benchmark			Result	
Abbreviation	Full Form	Terrible	Acceptable	Excellent	Value	Interpretation
CMIN/df	Chi-Square to Degrees of Freedom Ratio	≥5	≥3	≥1	1.851	Excellent
CFI	Comparative Fit Index	≤0.90	<0.95	≥0.95	0.981	Excellent
GFI	Goodness-of-fit Index	n/a	≥0.90	≥0.95	0.951	Excellent
AGFI	Adjusted Goodness-of-fit Index	n/a	n/a	≥0.90	0.929	Excellent
NFI	Normalized Fit Index	<0.80	≥0.90	≥0.95	0.960	Excellent
TLI	Tucker–Lewis Index	n/a	n/a	≥0.95	0.976	Excellent
RMSEA	Root Mean Square Error of Approximation	≥0.08	>0.06	≤0.06	0.046	Excellent

**Table 6:** Hypotheses testing result.

#	Hypothesis	Hypothesized Path	Estimate	*p*-Value		Hypothesis Testing Result
				Value	Sig.	
1	H1	PeoU → PU	0.209	0.000	***	Supported
2	H2-a	AS → PU	0.127	0.015	*	Supported
3	H2-b	AS → PEoU	0.189	0.002	**	Supported
4	H3-a	ES → PU	0.080	0.160	0.160	Unsupported
5	H3-b	ES → PEoU	0.099	0.136	0.136	Unsupported
6	H4	AS → ES	0.443	0.000	***	Supported
7	H5-a	PR → AS	0.672	0.000	***	Supported
8	H5-b	PR → ES	0.283	0.000	***	Supported
9	H5-c	PR → PU	0.427	0.000	***	Supported
10	H5-d	PR → PEoU	0.469	0.000	***	Supported

*** *p* < 0.001, ** *p* < 0.01, * *p* < 0.05.

## Data Availability

Data of the current study are available on Figshare at https://doi.org/10.6084/m9.figshare.25036751.v1.
